# Small nucleolar RNAs: continuing identification of novel members and increasing diversity of their molecular mechanisms of action

**DOI:** 10.1042/BST20191046

**Published:** 2020-04-08

**Authors:** Danny Bergeron, Étienne Fafard-Couture, Michelle S. Scott

**Affiliations:** Département de Biochimie et Génomique Fonctionnelle, Faculté de médecine et des sciences de la santé, Université de Sherbrooke, Sherbrooke, QC J1E 4K8, Canada

**Keywords:** gene annotation, gene expression regulation, RNA modification, RNA–protein binding, RNA–RNA binding, snoRNAs

## Abstract

Identified five decades ago amongst the most abundant cellular RNAs, small nucleolar RNAs (snoRNAs) were initially described as serving as guides for the methylation and pseudouridylation of ribosomal RNA through direct base pairing. In recent years, however, increasingly powerful high-throughput genomic approaches and strategies have led to the discovery of many new members of the family and surprising diversity in snoRNA functionality and mechanisms of action. SnoRNAs are now known to target RNAs of many biotypes for a wider range of modifications, interact with diverse binding partners, compete with other binders for functional interactions, recruit diverse players to targets and affect protein function and accessibility through direct interaction. This mini-review presents the continuing characterization of the snoRNome through the identification of new snoRNA members and the discovery of their mechanisms of action, revealing a highly versatile noncoding family playing central regulatory roles and connecting the main cellular processes.

## Introduction

Small nucleolar RNAs (snoRNAs) are an ancient and large family of noncoding RNAs present in all eukaryotes and a subset of archaea [1,2]. The best-characterized function of snoRNAs is in ribosome biogenesis, many snoRNAs serving as guides for the site-specific chemical modification of ribosomal RNA (rRNA). Two main families of snoRNAs have been described, the box C/D snoRNAs and the box H/ACA snoRNAs respectively catalyzing 2′-O-ribose methylation and pseudouridylation of their substrates. With ∼100–200 sites known to carry these modifications in rRNA, it was expected, two decades ago, that the complement of snoRNAs would number ∼200 in vertebrates [3]. However, diverse experimental and computational strategies have led to, and continue to result in the identification of novel snoRNAs, which now add up to between several hundreds and several thousands for many vertebrates, although not all have been shown to be expressed [4–6]. In parallel to the discovery of new members of the snoRNA family, the past decade has also led to the characterization of many diverse novel regulatory functions, affecting many cellular processes in addition to ribosome biogenesis [7]. These studies, many of them fortuitous, describe novel binding partners and molecular mechanisms, expanding both the targets of snoRNAs and their mechanisms of action. This mini-review describes the continuing expansion of the known snoRNome and of its functional capacity.

## The continuing identification of snoRNAs

**Figure 1.**
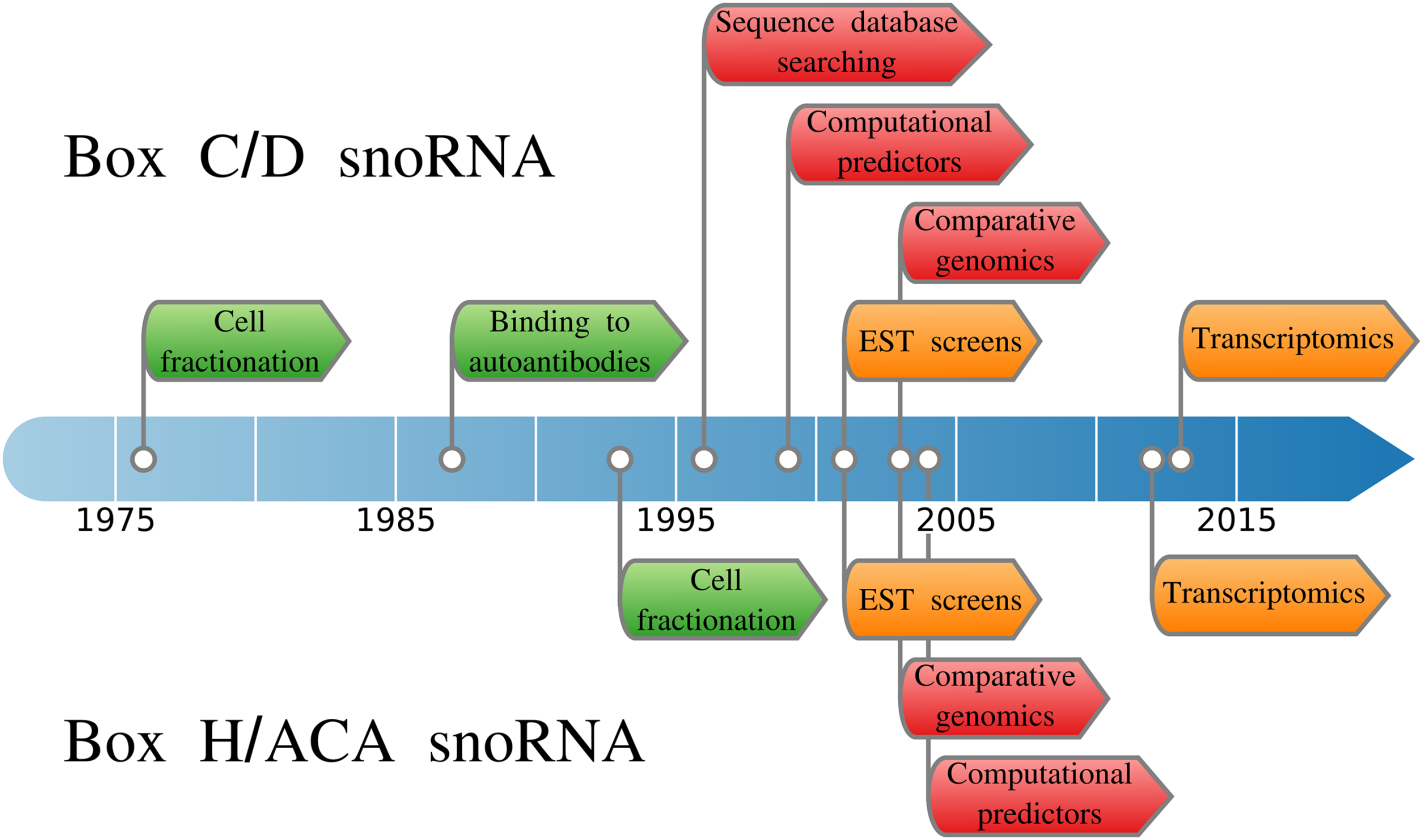
Timeline of the usage of different snoRNA identification strategies. The identification strategies are grouped into three categories: biochemical approaches in green, high-throughput strategies in orange and computational approaches in red. Box C/D and box H/ACA discoveries are described respectively above and below the timeline.

Over the past five decades, diverse strategies have been employed to identify snoRNAs, building on our knowledge of their characteristics and available technologies (Figure 1).

### Cell fractionation and binding to autoantibodies

**Figure 2.**
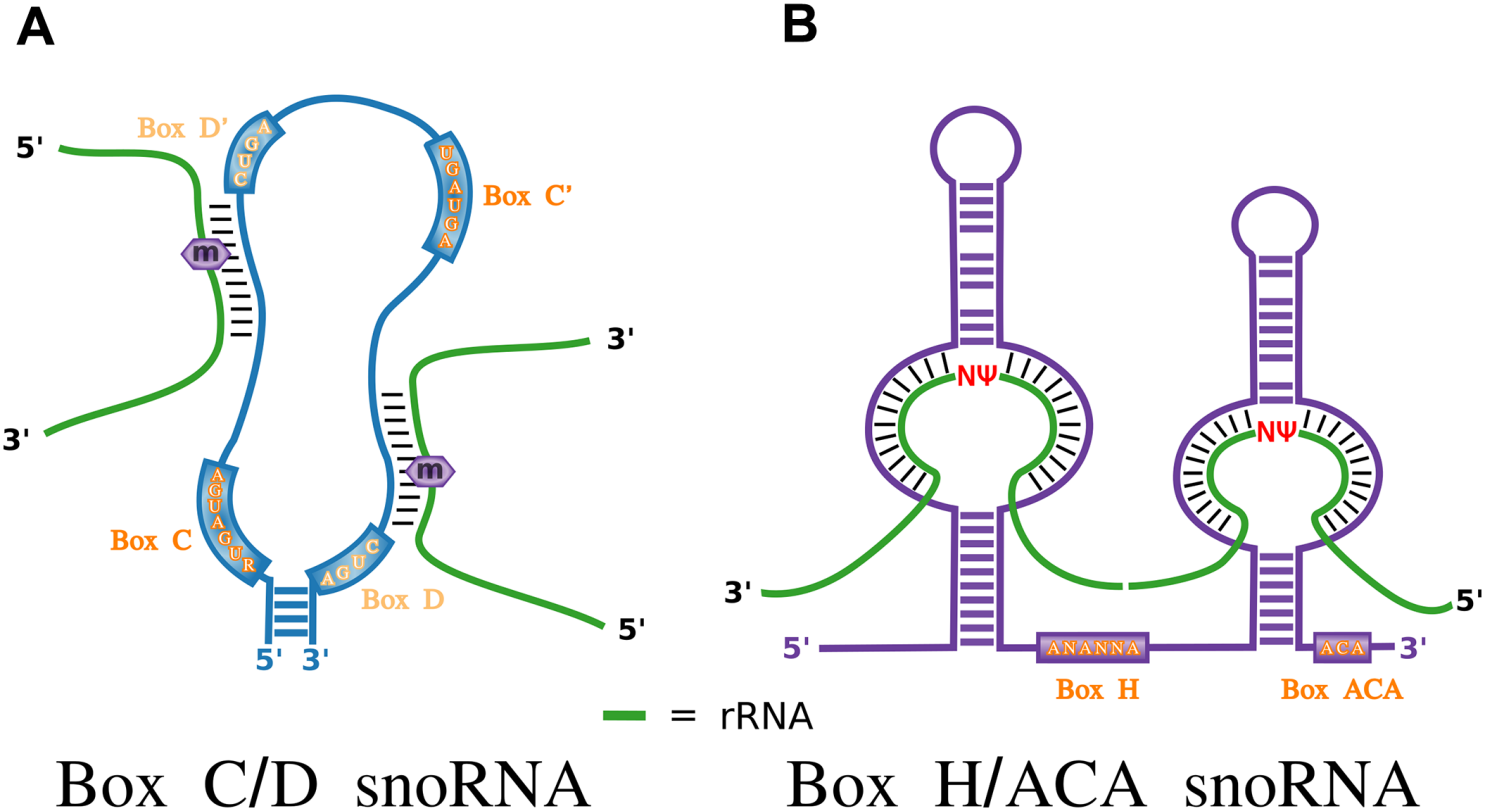
Canonical features of snoRNAs. (**A**) Canonical box C/D snoRNAs are characterized by the presence of a terminal stem, sequence motifs (boxes C, C′, D′ and D) and guide regions with complementarity to target sequences in rRNA. The target residue base pairing with the fifth nucleotide upstream of the box D′ or D is methylated (shown by the m in a purple hexagon). (**B**) Canonical H/ACA snoRNAs consist of two tight hairpins separated by a hinge region (box H) and terminated by an ACA box found 3 nucleotides before the 3′ end. The hairpins typically have a bulge which is where the region of complementarity to the rRNA target is located. The uridine residue that is pseudouridylated is represented by a red Ψ.

The first snoRNA to be detected, U3 (SNORD3), was identified by biochemical fractionation experiments as amongst the most abundant nuclear small RNAs in eukaryotic cells [8]. Subsequent studies found U3 to form a ribonucleoprotein complex targeted by autoantibodies from a patient with diffuse scleroderma [9]. The specific target of the autoantibodies was the box C/D snoRNA core-binding protein fibrillarin (FBL) and these antibodies enabled subsequently the identification of many other box C/D snoRNAs starting with U8 (SNORD118), U13 (SNORD13), U14 (SNORD14) and U15 (SNORD15) in human [10,11]. These early discoveries revealed common characteristics of box C/D snoRNAs which consist of a loose hairpin with a terminal stem, box C and box D sequence motifs positioned respectively near the 5′ and 3′ ends of the molecule (Figure 2A), strong conservation and in many cases intronic localization. In doing so, they provided the foundation for the discovery of other members of the family. In parallel, cell fractionation of the nucleus also led to the identification of a second family, later termed box H/ACA snoRNAs, also showing strong evolutionary conservation and intronic localization, but displaying different sequence motifs and binding to different proteins [12–15]. Box H/ACA snoRNAs are characterized by the presence of two tight hairpins, separated by a hinge region (box H) and terminated by a box ACA found 3 nucleotides from the 3′ end (Figure 2B). Further characterization of snoRNAs in diverse eukaryotes indicated that while many snoRNAs are located in the introns of longer genes and require the transcription of these host genes for their own expression, others, such as U3, are transcribed from their own promoters and contain a trimethylguanosine cap structure at their 5′ termini [2,16,17].

### Database searching

The elucidation of the molecular function and targets of known snoRNAs in guiding 2′-O-ribose methylation and pseudouridylation of rRNA [18,19] was not only a fundamental discovery for the understanding of the role of snoRNAs in cell biology, but it also provided an additional important characteristic to enable the identification of further family members. Indeed, these studies demonstrated that snoRNAs have functional guide regions that base pair with their rRNA targets, specifying the exact position requiring modification (illustrated in Figure 2). This knowledge enabled a new search strategy through sequence databases such as Genbank, based not only on consensus motifs and evolutionary conservation but also, the presence of guide regions complementary to rRNA (e.g. [20,21]). Further database searches also led to the identification of additional snoRNAs with complementarity to small nuclear RNAs (snRNAs) rather than rRNA, expanding the targets of snoRNAs [22]. It was later found that a related family of noncoding RNAs, the small Cajal body RNAs (scaRNAs) guide the methylation and pseudouridylation of the snRNAs transcribed by the RNA polymerase II, and only the U6 snRNA, transcribed by the RNA polymerase III, is modified by snoRNAs [23,24].

### Computational predictors

**Figure 3.**
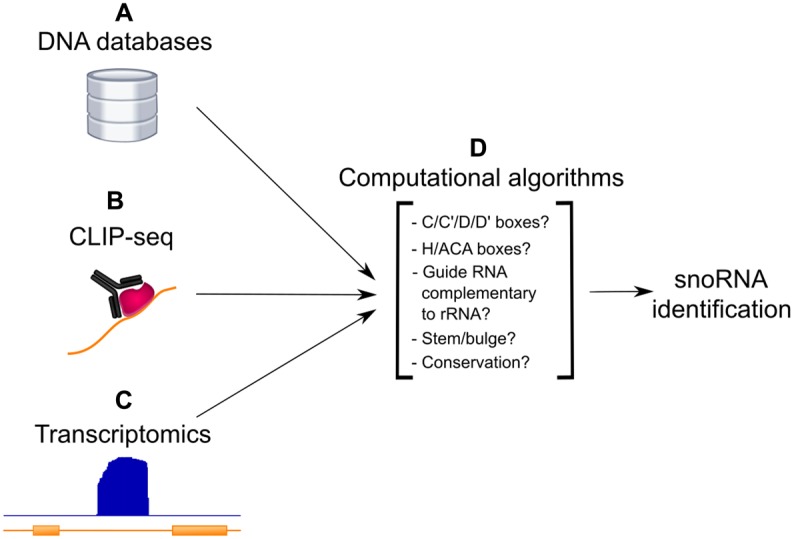
Computational approaches to identify snoRNAs. Starting from either sequence databases (**A**), the transcripts bound to specific proteins as detected by CLIP-seq experiments (**B**) or non-annotated genomic regions displaying strong levels of expression in RNA-seq datasets (**C**), computational algorithms consider several different features including, but not limited to, the presence of sequence motifs, complementarity to rRNA, secondary structure features and conservation to predict snoRNAs (**D**).

Knowledge of the main snoRNA characteristics, and the availability of whole genomes enabled the creation of computational predictors of snoRNAs, for both C/D and H/ACA as separate tools, first in yeast, then in other eukaryotes (e.g. [25–27]). Many of these studies predicted novel snoRNAs and then experimentally validated them. With increasing evidence of subsets of snoRNAs with non-canonical features including low evolutionary conservation, no known targets in rRNAs or snRNAs, and unusual genomic locations, computational screens progressively widened their scope and increased the number of annotated snoRNAs in diverse organisms (e.g. [28–31]). The different strategies to computationally predict snoRNAs have evolved over time, taking into consideration comparative genomics and transcriptomics datasets as described in the following sections, and are depicted in Figure 3.

### Large EST screens

Analysis of large-scale libraries of expressed sequence tags (ESTs) focusing on small RNAs (<500 nucleotides) provided unbiased screens of cellular transcripts and resulted in the identification of many novel snoRNAs. One of the first such screens, carried out in mouse, identified 201 novel snoRNAs, of both box C/D and H/ACA families [32]. Many of the novel snoRNAs had no identified targets and were labeled orphan snoRNAs, increasing the diversity of the snoRNome. Subsequent such screens in diverse organisms including human, fungi and the silkworm continued the identification of additional snoRNA members (e.g. [33–36]).

### Comparative genomics approaches

The comparison of snoRNA loci across genomes increased the number of annotated snoRNAs in many organisms. An early example of this strategy involved the computational prediction of snoRNA in the rice genome based on sequence and structural features followed by homology comparisons with Arabidopsis and corn genes, resulting in the identification of hundreds of rice snoRNAs [37]. Different subsequent comparative genomics approaches were employed including the computational comparative analysis of six yeast species [38], the comparison of orthologous introns across mammals [39], the study of the coevolution of snoRNAs and their rRNA and snRNA target sites across vertebrates [40] and an extensive comparative genomics analysis of fungal snoRNomes [41], all resulting in the discovery of novel members of the snoRNA family.

### Transcriptomics

The advent of high-throughput sequencing led to the identification of many novel snoRNAs. One such strategy involved immunoprecipitation of snoRNA binding proteins followed by sequencing. For example, hundreds of human members of a new subfamily of H/ACA snoRNAs, the AluACA RNAs derived from Alu intronic elements, were detected as bound to the Cajal body localization protein Wdr79 [42]. Similarly, the sequencing of RNA bound following photoreactive nucleotide-enhanced cross-linking and immunoprecipitation (PAR-CLIP) of core snoRNA-associated proteins NOP58, NOP56, FBL and dyskerin (DKC1) led to the identification of dozens of new human snoRNAs [43].

Transcriptomics datasets have also been used to identify expressed snoRNAs and to confirm snoRNA predictions. For example, ENCODE project small RNA-seq data were used to filter computational predictions of snoRNAs across the human genome, resulting in the identification of several dozen new snoRNAs [44]. SnoRNAs pose challenges in their accurate quantification, due to their size and stable structure. Several methodologies have attempted to address these issues, including an rRNA depletion strategy to enrich for non-rRNA in small RNA-seq datasets, resulting in the identification of over 100 new expressed snoRNA in the alga *Euglena gracilis* [45]. The use of reverse transcriptases from thermophilic bacteria has been shown to faithfully represent the abundance of highly structured RNAs such as transfer RNAs (tRNA) and snoRNAs [46,47]. Using thermostable group II intron reverse transcriptase sequencing on non-fragmented RNA samples, 25 non-annotated human snoRNAs were recently identified, including 22 box H/ACA snoRNA shown to be dependent on DKC1, the pseudouridine transferase H/ACA binding partner [4,48].

Thus over the past four decades, diverse strategies have enabled the identification of snoRNAs in many organisms, providing increasing insight into their characteristics and leading to their classification. It should be noted, however, that not all snoRNAs present in databases have been experimentally shown to be expressed and some might be inactive copies. Users of such resources should take this into consideration.

## Diversity of the mechanism of action of snoRNAs

**Figure 4.**
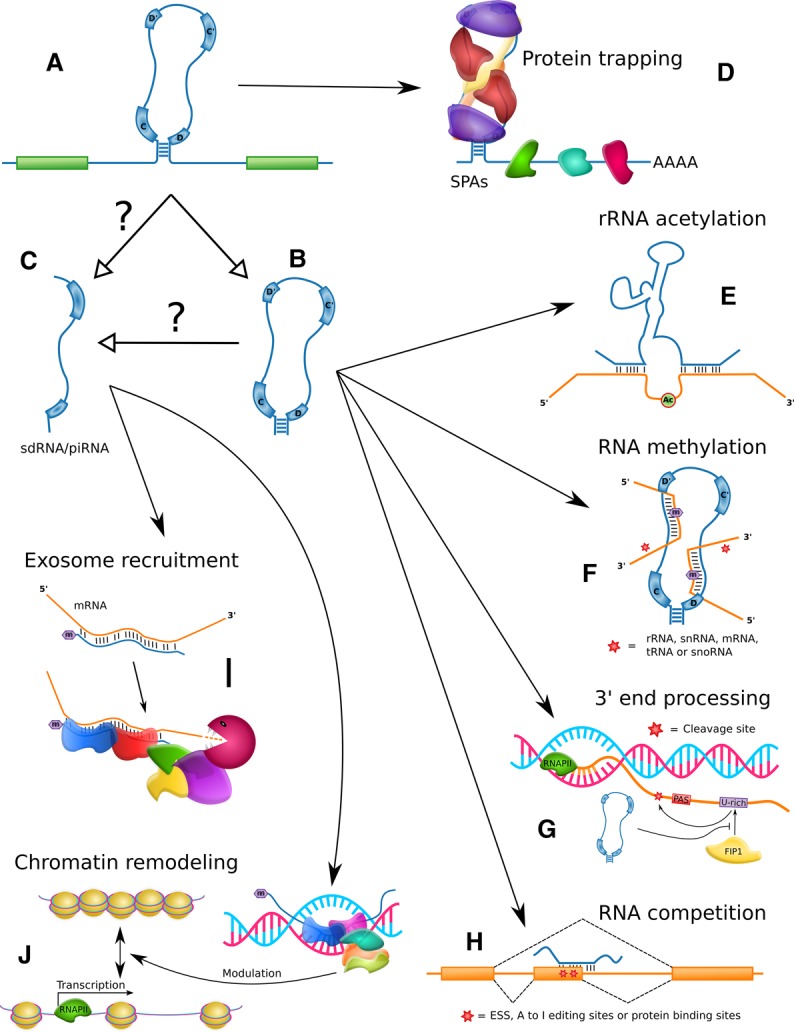
Overview of non-canonical mechanisms of action described for snoRNAs. (**A**) Mammalian snoRNAs are typically embedded in an intron of another gene. (**B**) Following splicing, intron debranching, protein binding and exonucleolytic degradation, the mature snoRNA is formed. (**C**) Stable fragments of snoRNAs referred to as sdRNAs for snoRNA-derived RNAs have been detected and could be processed from the mature snoRNA or its precursors. Some sdRNAs have been characterized as piRNAs. (**D**) Longer noncoding transcripts containing snoRNAs have been found to sequester specific proteins. (**E**) Some snoRNAs can acetylate rRNA. (**F**) SnoRNAs can methylate diverse non-canonical substrates including tRNA and mRNA. (**G**) Specific snoRNAs can bind 3′ end processing protein factors, affecting the choice of polyadenylation sites. (**H**) SnoRNAs can interact with other RNA, competing for functional binding sites. (**I**) SdRNAs can regulate pre-mRNA stability through direct binding and recruitment of the nuclear exosome. (**J**) SdRNAs can also recruit chromatin-modifying complexes to promoters by direct binding. Throughout the figure, white arrowheads indicate processing relationships whereas black arrowheads depict regulatory relationships.

Over the past two decades, the successive discoveries of novel snoRNAs and the identification of already annotated snoRNAs carrying out unexpected functions have led to the attribution of diverse new roles to snoRNAs. An excellent and extensive review of the diversity of snoRNA functions was recently published [7]. Strikingly, these recent studies also reveal the diversity that exists in the molecular mechanisms of action carried out by snoRNAs, from the chemical modification of RNA (with increasingly wide biotype range as substrates, from rRNA and snRNA to tRNA, protein_coding RNAs, snoRNAs and beyond) to binding competition, protein trapping and recruitment of protein factors to diverse targets (Figure 4). Here, we review some of the main highlights of snoRNA biology with a focus on their mechanism of action.

### Chemical modification of RNA

As described above, the best-characterized function of snoRNAs is to guide the site-specific modification of rRNAs and snRNAs. This canonical function is carried out through physical interaction between snoRNAs and their targets by Watson–Crick base pairing, bringing the target nucleotides to the catalytically active center of the FBL methyl transferase and the DKC1 pseudouridine synthase. However, variations of this functionality, including the type of modification, the enzyme involved and the biotype of the targets have been described.

#### Acetylation of canonical targets

Sharma et al. [49] revealed a mechanism by which two orphan yeast box C/D snoRNAs, snR4 and snR45, catalyze the acetylation of two cytosine residues of the 18S rRNA. Both snoRNAs use bipartite complementarity to the 18S rRNA to expose the cytosine to be modified, a mechanism reminiscent of canonical pseudouridylation by box H/ACA snoRNAs, and the associated enzyme carrying out the acetylation was shown to be Kre33 (Figure 4E).

#### Chemical modification of non-canonical RNA

Several independent studies have reported the capacity of some snoRNAs to guide the modification of RNAs other than rRNA or snRNA. For example, a *de novo* analysis of published FBL CLIP-seq datasets led Elliott and colleagues to identify the Pxdn messenger RNA (mRNA) which encodes an abundant peroxidase of the circulatory system, as an interactor of SNORD32A [50]. SNORD32A was previously identified amongst a small group of snoRNAs shown to regulate reactive oxygen species pathways and oxidative stress, but the molecular mechanism employed remained unknown. Strong sequence complementarity was found between Pxdn mRNA and SNORD32A as well as SNORD51 which has an almost identical antisense sequence as SNORD32A. Knockdown of FBL or SNORD32A + SNORD51 led to significant reduction in 2′-O-methylation of the Pxdn mRNA and to decreased levels of Pxdn mRNA but, surprisingly, increased levels of the protein. Further experiments demonstrated a reduced translation efficiency of the 2′-O-methylated mRNA compared with its unmodified counterpart.

Along the same lines, Vitali and Kiss [51] showed recently that tRNA could also be 2′-O-ribose methylation targets of snoRNA/scaRNA. Bioinformatics analyses in search of the function of the orphan box C/D snoRNA SNORD97 and its Cajal body homolog SCARNA97 (also known as SNORD133) led to the discovery of strong sequence complementarity between the two snoRNA guide regions and the tRNA^Met^(CAT) around its position C34, which was already known to be 2′-O-methylated. Only the depletion of both snoRNAs showed strong effects on this tRNA methylation, suggesting a redundancy of function for these two molecules. Digging deeper into the functional role of this modification, the authors reported that the C34 2′-O-methylation prevents the cleavage of the tRNA by the anticodon stress-induced endoribonuclease angiogenin. This cleavage leads to the production of 5′ and 3′ tRNA-derived fragments (tRF) that have been proposed to play regulatory roles.

Taken together, these examples demonstrate the diversity of both snoRNA targets (Figure 4F) and of their functional impact through their 2′-O-methylation capacity. Together with a recent report of the detection of hundreds of 2′-O-methylation sites in mRNAs in HeLa and HEK cells [52], and our increasing understanding of the role of this modification, including in RNA stability, splicing and translation (as reviewed in [53]), these studies raise questions about the possibility of widespread involvement of snoRNAs in these regulatory processes.

### Binding competition through RNA–RNA interactions

While the range of snoRNA modification targets continues to widen, the base pairing of snoRNAs does not always lead to modification of their interactors. Increasing numbers of examples suggest that the direct binding of snoRNAs to other RNAs could alternatively interfere with the natural binding of other interactors (Figure 4H).

One of the first examples of this type of molecular mechanism was the box C/D snoRNA SNORD115 and its implication in the alternative splicing of the serotonin receptor 2C gene (HTR2C) [54]. This regulatory mechanism has been extensively described elsewhere (as reviewed for example in [7,55]), but briefly, the orphan SNORD115 was shown to bind the alternative exon Vb of the HTR2C mRNA, the inclusion of which is necessary for functional activity of the resulting receptor. The binding of SNORD115 to the mRNA was shown to serve two main purposes: prevent exonic splicing silencer elements near this region from being bound by splicing regulator proteins and inhibit the A to I base editing of the mRNA [54,56–58]. Those two functions, which continue to be confirmed by more recent studies (e.g. [56]), are performed simply by masking partially a specific sequence of the mRNA, but have a huge impact on the receptor activity.

SNORD27 was also shown to influence alternative splicing, possibly through binding competition. A 2016 study from the Stamm group investigating the possible role of snoRNAs outside the nucleolus characterized a population of snoRNA complexes devoid of FBL [59]. Several box C/D snoRNAs including SNORD27, were found enriched in a subfraction of the nucleus containing mostly spliceosomal components. Using a genome-wide analysis for potential targets with sequence complementarity to SNORD27, E2F7 pre-mRNA was identified. Knockdown experiments of the snoRNA using antisense oligonucleotides (ASO) almost entirely abolished the alternative exon exclusion, while further minigene experiments with the replacement of the snoRNA targeting region indicated the requirement of a direct interaction, supporting the functional importance of SNORD27 in alternative splicing regulation [59]. In addition to E2F7, 8 of the 30 SNORD27 predicted targets showed alternative splicing patterns regulated by SNORD27, demonstrating a broad range of action of this snoRNA.

Interestingly, SNORD27 was already known to have a canonical role in guiding the methylation of 18S rRNA. The role of SNORD27 in the regulation of splicing involved its participation in a complex devoid of FBL, through its 3′ portion, possibly resulting in competition for the U1 snRNP binding site. These findings suggest that binding of the snoRNA to various target types (rRNA and mRNA) can use different portions of the snoRNA and involve different molecular mechanisms (reviewed in [55]).

### Other snoRNA–RNA interactions

Over the last five years, three groups developed similar protocols to map RNA–RNA interactions in a high-throughput manner. PARIS, SPLASH and LIGR-seq, all employ psoralen cross-linking, proximity ligation followed by deep sequencing and RNA-duplex characterization, detecting hundreds of thousands of RNA–RNA interactions [60–62]. While the percentage of mapped reads that are RNA chimeras is low, and even more so those involving snoRNAs, a few thousands of snoRNA–RNA interactions are detected including many snoRNA–rRNA, snoRNA–mRNA and snoRNA–snoRNA interactions. Interestingly, Sharma et al. [61] who designed the LIGR-seq protocol discovered three new non-canonical targets for the orphan box C/D snoRNA SNORD83B. SNORD83B knockdown led to increased levels of three mRNAs, NOP14, RPS5 and SRSF3, proving the potential of these methods for the discovery of non-canonical targets for snoRNAs.

While snoRNA–rRNA interactions have been extensively characterized and we are progressively gaining insight into the role of snoRNAs in mRNA fate, the role of snoRNA–snoRNA interactions and modification of snoRNA remain elusive even though several groups have detected them (e.g. [43]). As new non-canonical snoRNA functions emerge, it will be interesting to revisit these interactions/modifications to gain a bigger picture of the different metabolic pathways involved.

### Recruitment of protein factors

#### Recruitment of the nuclear exosome

Many groups have reported fragmented profiles of some snoRNAs in high-throughput sequencing analysis. These fragments were subsequently termed small nucleolar RNA-derived RNAs (sdRNAs) (e.g. [63–65]). While investigating the small RNA profile in primary CD4T-lymphocytes, Zhong et al. [66] found a highly expressed sdRNA, generated from SNORD63 processing, which was previously classified as piRNA piR30840. This snoRNA fragment was confirmed to interact with two known piRNA binding proteins, Piwil4 and Ago4, and to carry a 2′-O-methyl group at its 3′ end, two classical features of piRNAs [67]. The overexpression of this sd/piRNA in CD4T-lymphocytes resulted in a decreased expression of some cytokines including IL-4 which has a region of complementarity to the sdRNA in intron 2 of the gene. Both Piwil4 and Ago4 were subsequently shown to be necessary for IL-4 down-regulation by piR30840 through the recruitment of the Trf4/Air2/Mtr4 polyadenylation (TRAMP) complex and the nuclear exosome. These results strongly suggest that snoRNAs can be processed into smaller piRNA-like molecules that can have important functions notably in targeting mRNAs to degradation by the exosome complex (Figure 4I).

#### Recruitment of chromatin-modifying complexes

Similarly to the previous example, a recent study found additional piRNA-like molecules derived from snoRNAs encoded in introns of the lncRNA Growth Arrest Specific 5 (GAS5) [68], including one produced from SNORD75. This specific RNA named pi-sno75 was shown to have genuine piRNA features such as binding to PIWI proteins and a 3′ terminal 2′-O-methylation. Interestingly, the pi-sno75 was found to bind the promoter of the TNF-related apoptosis-inducing ligand (TRAIL) gene. With the help of PIWI proteins and other cofactors, the piRNA-like molecule was further shown to recruit both methyltransferase and demethyltransferase complexes to induce chromatin remodeling near the binding site, which resulted in an increased transcription of the gene [68]. To our knowledge, this is the first example of a snoRNA-processed RNA that targets DNA and plays a role in the regulation of the chromatin state (Figure 4J), although previous studies have identified an enrichment of snoRNAs in chromatin-associated RNAs (e.g. [69]). While the scope of action of these processed snoRNAs is unknown, based on these two previous examples, it is tempting to think that this is a widespread mechanism of gene regulation in the cell.

### Binding competition, protein activation and protein trapping through RNA–protein interactions

Increasing numbers of reports identify snoRNAs interacting with proteins that differ from the core snoRNA binding proteins known to be involved in canonical functionality. Several different molecular mechanisms have been teased out. For example, SNORD50A and SNORD50B were shown to bind on GTPases of the Ras superfamily such as K-Ras [70]. These snoRNAs were found to block access of farnesyltransferases to K-Ras, therefore, preventing the farnesylation of this GTPase. In the case of many cancers, the *SNORD50A–SNORD50B* locus was found to be deleted, leading to an enhanced prenylation of K-Ras [70]. The addition of the hydrophobic farnesyl group on K-Ras is critical for its association with the plasma membrane and thus its function to activate the subsequent ERK1/2 proliferation pathway [71]. In other words, by binding to Ras GTPases, SNORD50A and SNORD50B suppress their oncogenic functions.

Another interesting example is the interaction of snoRNAs and PARP-1 or poly(ADP-ribose) polymerase 1, a nuclear enzyme implicated in DNA damage repair. The inhibition of PARP-1 is helpful in the treatment of cancers because it causes accumulation of DNA damage and synthetic lethality of cancer cells presenting defects in homologous recombination (HR)-mediated DNA repair machinery [72]. However, even tumors with non-mutated HR machinery were recently shown to be affected by PARP inhibitors [73], and Kim et al. [74] discovered a mechanism that can explain this. Indeed, several mature H/ACA box snoRNAs (notably SNORA73A, SNORA73B and SNORA74A) can bind to PARP-1, leading to the activation of its ADPRylation (PAR) function. PARP-1 can then ADP-ribosylate the helicase DDX21, thereby promoting DDX21 localization to nucleoli and rDNA transcription. This, in turn, leads to enhanced ribosome biogenesis, protein translation and cell proliferation. ASO-mediated knockdown of SNORA74A engendered a substantial decrease in DDX21 ADPRylation and cell proliferation, confirming that snoRNAs can activate PARP-1 in the absence of DNA damage. It was also shown that DDX21 can bind snoRNAs such as SCARNA2 on its C-terminal RNA-binding domain [74]. These snoRNAs were suggested to compete with the PARP-1 and DDX21 interactions. Thus, snoRNAs function both as activators and as competitive binders in this cellular pathway.

In another example, several snoRNAs were recently shown to interact with components of the cleavage and polyadenylation specificity factor (CPSF), thereby modulating mRNA 3′ end processing [75]. These snoRNAs were not associated with canonical snoRNP core proteins, as demonstrated by western blots. One of the reported snoRNA, SNORD50A, bound to FIP1 *in vivo* and impeded its interaction with polyA sites. This resulted in the reduction in mRNA 3′ processing of various targets, notably many mRNAs transcribed from genes involved in cell proliferation and apoptosis. The depletion of SNORD50A with ASO led to enhanced mRNA 3′ processing and up-regulation of the expression of the corresponding genes, confirming that SNORD50A acts as a negative regulator of mRNA 3′ end processing (Figure 4G).

Interestingly, while smaller fragments of snoRNAs play functional roles in cells as described above, longer precursors of snoRNAs can also serve to regulate specific processes. In recent years, several research groups found that snoRNAs retained in longer RNAs could act as decoys for proteins, sequestering them away from their normal cellular localization and at the same time hindering their activity (Figure 4D).

For example, Lykke-Andersen et al. [76] reported that SNORD86, a snoRNA encoded within the *NOP56* pre-mRNA, acted in *cis* to control the levels of the C/D snoRNP core protein NOP56 depending on the availability of different snoRNP proteins. SNORD86 was shown to adopt two alternate conformations. If the level of proteins involved in the snoRNP assembly complex was low, the snoRNA adopted the ‘non-snoRNP’ structure, thereby activating the upstream splice donor (uSD) and repressing the downstream splice donor (dSD), resulting in the excision of the intron harboring SNORD86 and the formation of a protein-coding NOP56 mRNA and its subsequent translation. Conversely, if the level of snoRNP core proteins was in excess in the nucleus, such proteins bound to SNORD86, forcing it to adopt the ‘snoRNP’ conformation, de-activating and de-repressing the uSD and dSD, respectively, resulting in the retention of the SNORD86 intron. The resulting transcript was processed by the nonsense-mediated decay machinery in the cytoplasm, generating a fragment protected from exonucleolysis by the snoRNP, and sequestering proteins such as FBL and NOP56 out of the nucleus where their excess concentration could lead to ribosomal biogenesis defects.

Another interesting group of longer RNA containing snoRNAs are the sno-lncRNAs of the Prader–Willi syndrome (PWS) region which harbors not only the SNORD115 and SNORD116 clusters but also lncRNAs bordered by snoRNAs on their ends (sno-lncRNAs) and 5′-snoRNA-capped and 3′-polyadenylated lncRNAs (SPAs) [77]. It has been recently shown by individual-nucleotide resolution UV crosslinking and immunoprecipitation (iCLIP) analysis that SPA1 and SPA2, two SPAs located in the 3′UTR of the SNURF–SNRPN locus of the PWS region, could interact with three RNA-binding proteins (RBPs) via the snoRNA contained in their 5′ end (SNORD107 and SNORD109A, respectively). More than 1% of TDP43, RBFOX2 and hnRNP M RBPs were reported to be sequestered by these SPAs in nuclear accumulations [77]. SPA1 and SPA2 are absent from PWS patients, thereby leading to an increased number of RBPs available for alternative splicing. It is thus not surprising that induced pluripotent stem cells (iPSC) derived from PWS patients present altered splicing patterns in various mRNA, some of which are involved in synapse function. Similarly, sno-lncRNAs encoded in the PWS region were also reported to interact with protein members of the Fox family, notably RBFOX2, thereby trapping these RBPs and impeding their splicing regulatory functions [78] and a subset of box C/D snoRNAs were shown to depend on RBFOX2 for their accumulation [79]. These data and others demonstrate an extensive and complex relationship between snoRNAs, splicing factors, the spliceosome and specific alternative splicing events (reviewed in [55,80–82]).

## Perspectives

- Overall, recent years have witnessed a considerable increase in the number of snoRNAs annotated in genomes thanks to our diversifying toolkit enabling their identification. This increase in our capacity to detect snoRNAs has been accompanied by numerous, often surprising discoveries of the versatility of snoRNA roles and mechanisms of action and of their wide implication in cellular functionality.
- *Importance in the field*: SnoRNAs are stable and abundant noncoding RNA implicated in the regulation of a plethora of cellular processes as witnessed by the ever-increasing number of studies describing their non-canonical functions. The wide range of their mechanisms of action presented in this review is likely only the tip of the iceberg.
- *Current thinking:* SnoRNAs are currently often overlooked, amongst other reasons due to technical difficulties in their detection, but despite these challenges, recent literature increasingly provides evidence of their central role in cells and their involvement in diseases.
- *Future directions:* Many snoRNAs remain annotated as orphans, for which cellular functionality is unknown and snoRNAs can carry out more than one function, making them highly versatile. The extent of functionality for all snoRNAs and the interplay between their different functions will be important to decipher. In addition, most non-canonical functions of snoRNAs have been described for box C/D snoRNAs, which could be due to the increased difficulty in detecting and accurately quantifying box H/ACA snoRNAs. Their characterization will likely lead to many more unexpected discoveries. The next decade will likely uncover even more diverse examples and mechanisms of snoRNA capacities but will also provide integrative studies increasing our understanding of the snoRNome as a key component and master regulator connecting the main fundamental processes of the cell.

## Abbreviations

ASO: antisense oligonucleotides
dSD: downstream splice donor
ESTs: expressed sequence tags
mRNA: messenger RNA
PWS: Prader–Willi syndrome
RBPs: RNA-binding proteins
rRNA: ribosomal RNA
snoRNAs: Small nucleolar RNAs
snRNAs: small nuclear RNAs
tRF: tRNA-derived fragments
uSD: upstream splice donor
